# On the Mercurial Treatment of Fever

**Published:** 1829-04-01

**Authors:** 


### NEW YORK HOSPITAL.

### On the Mercurial Treatment of Fever. By Dr. Brown.

From a long and able paper on typhus fever, published in our very respected cotemporary, the American Medical Recorder, by Dr. Brown, one of the physicians to the above hospital, which paper gained the prize of a silver-cup, we extract the following passage relative to the mercurial treatment of fever.—

"During my attendance at the New York hospital, in the months of September and October of last year, numerous cases of fever of different types, were received into that institution, from different parts of the country. Several of these cases occurred in seamen from southern parts, and assumed a character of a high bilious cast, some even bordering upon *yellow fever*. Several were cases of marsh fever, in labouring men from the interior, who had been employed upon the Morris canal in New Jersey, and in marshy districts in the state of New York. A number were cases of typhus, from among the poor of the city and neighbouring country.

"In many of the cases, the lancet was deemed necessary; and in some, emetics and blisters were found serviceable; but in all, calomel in full doses was used in the course of the treatment. In some of them of a very malignant character, I used calomel in ʒss. doses, once, and sometimes twice, in twenty-four hours; followed by some other cathartic after a lapse of six or eight hours, until free evacuations were procured. It was a common practice with me to prescribe it in fifteen and twenty grain doses. In addition to the calomel, followed by purgatives, other means were used, as the warm bath, blisters, diaphoretics, &c. according to circumstances. Under this course, cases recovered which were very malignant, and some which were to all appearance desperate. Calomel in full doses, (ten to twenty grains,) produces an impression in fevers, which cannot be accomplished by any other single or combined cathartic. It excites more surely and generally, the secretions, than any other, and with less expenditure of the energies of the system. The liver, pancreas, stomach, and bowels, all inactive or paralyzed, are at once aroused by its resistless impulse; which is immediately manifested by the consistent discharges which always follow its administration in full doses. And the effect is not confined here.—The skin and kidneys, if not excited by the calomel itself to increased secretion, are more easily acted upon by diaphoretics and diuretics, through its influence. While other cathartics, with various efficacy, bring away watery discharges from the stomach and bowels, which exhaust the strength of the patient, without striking, as it were, at the seat and foundation of the disease, calomel acts specifically upon the liver, spleen, pancreas, &c. re-animating the suppressed functions of these organs, without which, very little is gained over diseased action. If an action can be thus maintained upon these organs, the progress of the disease may surely be kept within safe limits, the equilibrium of the circulation restored, and a final solution of febrile action produced.

"The favourable effect of calomel in full doses, followed by other cathartics, may be observed in the appearance of a clammy secretion about the mouth and fauces. I have often observed within twelve hours after the exhibition of ten or fifteen grains, a viscid secretion and moisture about the tongue and fauces, in cases where there before existed a complete husky dryness of these parts. A second dose would produce a moisture, whitening, and softening of the edges of the tongue. The daily continuance of the practice, discovers the change in the appearance of this organ, proceeding from the root and edges, to the centre, and finally the apex (which shews the evidence of diminished secretion longer than any other part of the tongue,) becomes moist, soft, and of a natural complexion. In some very obstinate cases, I have remarked, that this moisture of the edges of the tongue, was maintained for many days in succession, by the daily administration of calomel, without any thing, apparently, having been gained; when, finally, the redness and dryness of the organ would begin to yield more and more, until the whole would assume a softness, with moisture, and a pale natural hue. At the same time, the secretions, in general, would be more unlocked, by the same degrees, as the change in the appearance of the tongue, until a general restoration took place, with a more or less complete crisis. It is often several days after the restoration of the secretions, before the pulse comes fully down to the healthy standard in frequency; and until this point is gained, there is not much improvement of the appetite. Under these circumstances, tonics are seldom necessary, and I am confident, from my own observation, that, in the majority of cases, the patient does better without them."

The alterative effects were not the less obtained, in Dr. Brown's practice, because the medicine was administered in full doses. A slight tenderness of the gums and glands of the throat, with a moderate flow of saliva, was all that he observed to result from the doses of mercury above-mentioned. These symptoms were invariably followed by an abatement or "speedy solution of all fever."

